# High species diversity of the soft coral family Xeniidae (Octocorallia, Alcyonacea) in the temperate region of Japan revealed by morphological and molecular analyses

**DOI:** 10.3897/zookeys.862.31979

**Published:** 2019-07-09

**Authors:** Tatsuki Koido, Yukimitsu Imahara, Hironobu Fukami

**Affiliations:** 1 Interdisciplinary Graduate School of Agriculture and Engineering, University of Miyazaki, Gakuen-kibanadai-nishi-1-1, Miyazaki, 889-2192, Japan; 2 Biological Institute on Kuroshio, Kuroshio Biological Research Foundation, 560 Nishidomari, Otsuki, Kochi 788-0333, Japan; 3 Wakayama Laboratory, Biological Institute on Kuroshio, 300-11 Kire, Wakayama, 640-0351, Japan; 4 Department of Marine Biology and Environmental Sciences, Faculty of Agriculture, University of Miyazaki, Gakuen-kibanadai-nishi-1-1, Miyazaki, 889-2192, Japan

**Keywords:** high latitude, Miyazaki, phylogeny, taxonomy, Xeniidae

## Abstract

The soft coral family Xeniidae, commonly found in tropical and subtropical regions, consists of 20 genera and 162 species. To date, few studies on this family have been conducted in Japan, especially at higher latitudes. Although molecular phylogenetic analyses have recently been used to distinguish soft coral species, it is difficult to identify species and genera in this family due to the limited taxonomic indices and high morphological variation. In this study, we found a large Xeniidae community off the coast of Oshima Island (31°31.35'N, 131°24.27'E) at Miyazaki, Kyushu Island, located in the temperate region of Japan. The species composition and molecular phylogenetic relationships were investigated to uncover the species diversity of Xeniidae in this community. A total of 182 xeniid specimens were collected and identified to the species level, after which the samples were molecularly analyzed using a mitochondrial marker (ND2) and a nuclear marker (ITS) to infer the phylogenetic relationships. A total of 14 xeniid species were identified, including five undescribed species from five genera (*Anthelia*, *Heteroxenia*, *Sympodium*, *Xenia*, and *Yamazatum*). Miyazaki was identified as having the highest xeniid species diversity in Japan. The molecular phylogenetic trees inferred from each marker recovered very similar topologies: four genera (*Anthelia*, *Heteroxenia*, *Sympodium*, and *Yamazatum*) were monophyletic, whereas one (*Xenia*) was polyphyletic. Thus, except for *Xenia*, the morphological characteristics used for traditional taxonomy well reflected the phylogeny of the Xeniidae at the genus level. On the other hand, our results show that further taxonomic revisions of *Xenia* are needed.

## Introduction

Tropical marine animals, including zooxanthellate alcyonacean corals (i.e., soft corals) abound in the southern part of the temperate region of Japan, due to the Kuroshio – a strong warm current running along the coast from the Ryukyu Archipelago to the mainland of Japan. However, studies looking into the zooxanthellate alcyonacean corals in Japan are limited, especially regarding the family Xeniidae Ehrenberg, 1828. Xeniidae comprises 20 genera and 162 species (Cordeiro et al. 2019), and is distributed mainly across the Red Sea and the Indian and Pacific Oceans. Additionally, a few species have been found in the south Atlantic (Kükenthal 1906) and Norwegian Sea (Koren and Danielssen 1883; Danielssen 1887; Grieg 1887; Jungersen 1892). Although 21 species from seven genera in this family have been recorded in Japan (Utinomi 1950, 1955, 1958; Imahara 1996; Benayahu 2010), its current species diversity remains unknown due to the lack of recent surveys.

The present study describes a large community of xeniids found around Oshima Island (31°31.35'N, 131°24.27'E) at Miyazaki, Kyushu Island. As xeniids are uncommon in Japan, this is an unusual community. Due to this area’s higher latitude, coral reef structures are usually not formed, but there are over 100 zooxanthellate scleractinian coral species (Nishihira and Veron 1995). This area was occupied previously by zooxanthellate scleractinian corals, which were damaged drastically during the 1980s by outbreaks of the coral-eating gastropod *Drupella* spp. and the crown-of-thorns seastar *Acanthaster* sp. (Takayama and Shirasaki 1990). Currently, the area is occupied mainly by xeniids, which are known as pioneer alcyonaceans in ecological succession in tropical coral reefs (Benayahu and Loya 1987). Therefore, the ecological context of Oshima Island may represent an initial stage of secondary succession, following the drastic reduction of zooxanthellate scleractinian corals in the coral community. Identifying xeniid species diversity in this area is important to understand how coral communities change over time at higher latitudes.

Species identification difficulties are common among the anthozoans due to their limited key taxonomic characteristics and high morphological variation and plasticity. Recently, molecular phylogenetic analyses have been used to overcome such limitations. In particular, molecular phylogenetic data have been used frequently in scleractinian corals to revise taxonomy, identify cryptic species and describe new species (e.g., Budd et al. 2012; Huang et al. 2014a, b). In the alcyonacean corals, molecular phylogenetic analyses have also been applied to several families (France and Hoover 2002; McFadden and Hutchinson 2004; McFadden et al. 2009). For example, two genera, *Sphaerasclera* McFadden & van Ofwegen, 2013 and *Parasphaerasclera* McFadden & van Ofwegen, 2013 and the family Parasphaerascleridae McFadden & van Ofwegen, 2013 were described based on the results of combined molecular phylogenetic and morphological analyses (McFadden and Ofwegen 2013). For xeniids, molecular phylogenetic analyses have been also performed at the genus level. Haverkort-Yeh et al. (2013) used molecular phylogenetic trees with mitochondrial (COI, mtMutS) and nuclear (ITS, ATPSα) markers, to show that *Anthelia* Lamarck, 1816, *Heteroxenia* Kölliker, 1874, and *Sympodium* Ehrenberg, 1834 were genetically distinguishable from each other, whereas *Ovabunda* Alderslade, 2001 and *Xenia* Lamarck, 1816 were not. McFadden et al. (2014) also showed that *Anthelia*, *Cespitularia* Milne Edwards & Haime, 1850, and *Efflatounaria* Gohar, 1939 were genetically distant from all other xeniid genera, whereas *Ovabunda*, *Heteroxenia*, *Sansibia* Alderslade, 2000, and *Sarcothelia* Verrill, 1928 were paraphyletic with *Xenia* (COI, mtMutS, ND2, 28S rDNA). On the other hand, few molecular phylogenetic analyses have been performed in xeniids at the species level.

To date, the molecular data of xeniids indicate that mitochondrial ND2 marker is one of the best markers to infer the phylogenetic relationships among genera within many octocoral families (e.g., McFadden et al. 2006; McFadden et al. 2014), whereas nuclear ITS is a suitable marker to examine the octocorals’ species-level relationships. In particular, the ITS marker has been used to investigate the relationships between closely related species in the soft coral genera such as *Alcyonium* Linnaeus, 1758 and *Pseudopterogorgia* Kükenthal, 1919 (McFadden et al. 2001; McFadden and Hutchinson 2004; Sánchez et al. 2007; Dorado and Sánchez 2009). The present study aimed to investigate the current species diversity of the family Xeniidae around Oshima Island, Japan, and to clarify this family’s taxonomic issues at the species level, through molecular phylogenetic analyses using ND2 and ITS markers.

## Materials and methods

### Collection and identification of Xeniidae

**Figure 1.**
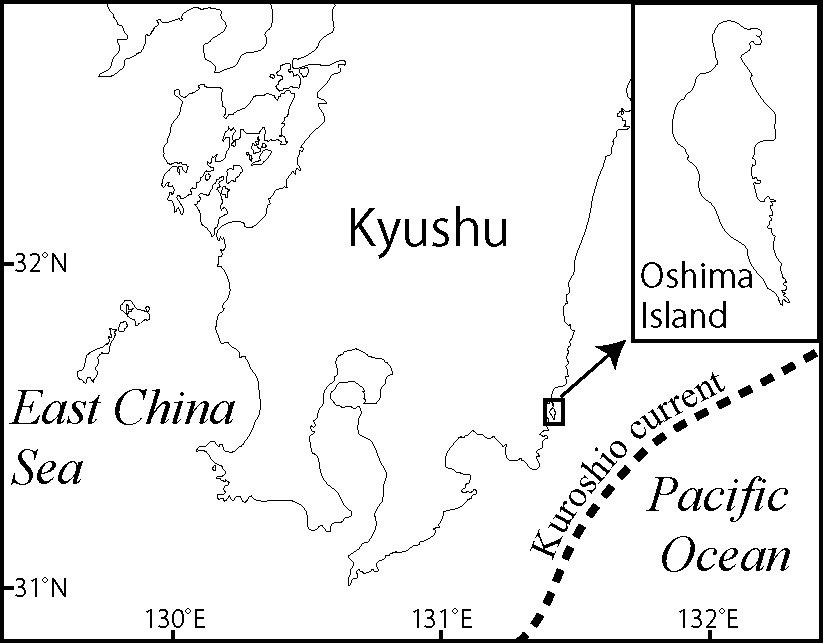
Map of the sampling sites of specimens of Xeniidae.

Specimens of xeniids were collected around Oshima Island, Miyazaki, Japan (31°31.35'N, 131°24.27'E; Fig. 1) by SCUBA or snorkeling. A small piece of tissue (5–10 mm) from each specimen was put into CHAOS solution (sterile distilled water 100 ml, guanidine thiocyanate 50 g, N-lauroyl sarcosin sodium 0.5 g, 1M Tris pH8 2.5 mL, 2-mercaptoethanol 0.7 mL) (Fukami et al. 2004) for molecular analyses, and the remaining portions of specimens were preserved in 99% ethanol for morphological analyses.

### Species identification

For species identification, we first summarized the morphological characteristics for all species in the five genera we found in this study (*Xenia*, *Heteroxenia*, *Sympodium*, *Yamazatum* Benayahu, 2010, and *Anthelia*) from original descriptions and related references to define the criteria for each species (Suppl. materials 1–5: Tables S1–S5), and used the summary to identify specimens at the species level. Table 1 shows a list of all specimens collected in this study. All specimens are deposited at Miyazaki University, Fisheries Sciences (MUFS) for coral collections (-C). Regarding specimen identification, the following morphological characteristics were measured or counted under stereo microscope: colony height, length and width of stalk, presence of branches, length and width of polyp, length and width of tentacle, length and width of pinnule, number of rows of pinnules, number of pinnules in the aboral row, sclerites form and sclerites size. In addition, microstructure of sclerites was observed by scanning electron microscope (SEM) (HITACHI Tabletop Microscope TM1000) as this morphological trait has been used recently to separate xeniid species (Janes and Mary 2012).

**Table 1.** Octocoral specimens for which partial ND2 and ITS sequences were obtained. MUFS-C: Miyazaki University, Fisheries Science for coral collections. NA: Not Analyzed.

Family	Species	Specimen Catalog #	Date	Depth (m)	GenBank #	
					ND2	ITS
Xeniidae	Anthelia cf. glauca	MUFS-COMO18	2012.7.2	4.3	LC467016	NA
		MUFS-COMO67	2012.12.25	<10	LC467017	LC467102
		MUFS-COMO70	2012.12.25	<10	LC467018	NA
	* Anthelia rosea *	MUFS-COTUN6	2014.12.3	<15	LC467019	LC467103
	Anthelia cf. tosana	MUFS-COMO13	2012.7.2	<5	LC467020	NA
	Heteroxenia cf. elisabethae	MUFS-COSU2	2012.5.5	<1	LC467021	LC467104
		MUFS-COSU3	2012.5.5	<1	LC467022	LC467105
	* Heteroxenia medioensis *	MUFS-COOTUC4	2014.12.3	<15	LC467023	LC467106
		MUFS-COOTUE3	2014.12.3	<15	LC467024	LC467107
	* Heteroxenia minuta *	MUFS-COMO10	2012.7.2	3.7	LC467025	LC467108
		MUFS-COMO12	2012.7.2	5.0	LC467026	LC467109
		MUFS-COMO28	2012.8.31	<10	LC467027	LC467110
	*Sympodium* sp. 1	MUFS-COMO63	2012.12.25	<10	LC467028	LC467111
		MUFS-COOTUG2	2014.12.3	<15	LC467029	LC467112
		MUFS-COOTUK16	2014.12.3	<15	LC467030	LC467113
	*Sympodium* sp. 2	MUFS-COMO149	2013.7.30	<10	LC467031	LC467114
	*Xenia* sp. 1	MUFS-COMO100	2012.12.25	<10	LC467032	LC467115
		MUFS-COMO154	2013.7.30	<10	LC467033	LC467116
		MUFS-COMO166	2013.7.30	<10	LC467034	LC467117
		MUFS-COMO4	2012.7.2	<5	LC467035	LC467118
		MUFS-COMO53	2012.12.25	<10	LC467036	LC467119
		MUFS-COMO54	2012.12.25	<10	LC467037	LC467120
		MUFS-COMO64	2012.12.25	<10	LC467038	LC467121
		MUFS-COMO68	2012.12.25	<10	LC467039	LC467122
		MUFS-COMO76	2012.12.25	<10	LC467040	LC467123
		MUFS-COMO77	2012.12.25	<10	LC467041	LC467124
		MUFS-COMO82	2012.12.25	<10	LC467042	LC467125
		MUFS-COMO83	2012.12.25	<10	LC467043	LC467126
		MUFS-COMO85	2012.12.25	<10	LC467044	NA
	* Xenia kuekenthali *	MUFS-COMO11	2012.7.2	2.9	LC467045	NA
		MUFS-COMO3	2012.7.2	3.9	LC467046	LC467127
		MUFS-COMO87	2012.12.25	<10	LC467047	NA
		MUFS-COMO152	2013.7.30	<10	LC467048	NA
	* Xenia novaecaledoniae *	MUFS-COMO155	2013.7.30	<10	LC467049	NA
		MUFS-COMO5	2012.7.2	3.2	LC467050	LC467128
		MUFS-COMO65	2012.12.25	<10	LC467051	LC467129
	* Xenia plicata *	MUFS-COKMG3	2014.12.3	<3	LC467052	NA
		MUFS-COMO148	2013.7.30	<10	LC467053	LC467130
		MUFS-COMO15	2012.7.2	4.6	LC467054	LC467131
		MUFS-COMO2	2012.7.2	4.8	LC467055	LC467132
		MUFS-COMO26	2012.8.31	<10	LC467056	NA
		MUFS-COMO40	2012.12.25	<10	LC467057	LC467133
		MUFS-COMO50	2012.12.25	<10	LC467058	LC467134
		MUFS-COMO69	2012.12.25	<10	LC467059	LC467135
		MUFS-COMO7	2012.7.2	4.0	LC467060	LC467136
		MUFS-COMO80	2012.12.25	<10	LC467061	LC467137
Xeniidae	*Xenia* sp. 2	MUFS-COMO161	2013.7.30	<10	LC467062	NA
		MUFS-COMO165	2013.7.30	<10	LC467063	LC467138
		MUFS-COMO6	2012.7.2	4.2	LC467064	NA
		MUFS-COMO8	2012.7.2	3.2	LC467065	LC467139
		MUFS-COMO9	2012.7.2	3.9	LC467066	LC467140
		MUFS-COSU1	2012.5.5	<1	LC467067	NA
		MUFS-COSU4	2012.5.5	<1	LC467068	LC467141
		MUFS-COSU5	2012.5.5	<1	LC467069	NA
		MUFS-COSU6	2012.5.5	<1	LC467070	NA
	*Yamazatum* sp. 1	MUFS-COMO1	2012.7.2	4.7	LC467071	LC467142
		MUFS-COMO14	2012.7.2	3.7	LC467072	LC467143
		MUFS-COMO147	2013.7.30	<10	LC467073	NA
		MUFS-COMO162	2013.7.30	<10	LC467074	NA
		MUFS-COMO42	2012.12.25	<10	LC467075	NA
		MUFS-COMO45	2012.12.25	<10	LC467076	NA
		MUFS-COMO48	2012.12.25	<10	LC467077	NA
		MUFS-COMO73	2012.12.25	<10	LC467078	NA
		MUFS-COMO89	2012.12.25	<10	LC467079	NA
Briareidae	*Briareum* sp.	MUFS-COMO17	2012.7.2	3.7	LC467080	NA
Clavulariidae	*Clavularia* sp.	MUFS-COAK6	2012.6.5	<3	LC467081	NA
Alcyoniidae	* Cladiella pachyclados *	MUFS-COSU13	2012.5.5	<1	LC467082	LC467144
	* Cladiella digitulatum *	MUFS-COSU14	2012.5.5	<1	LC467083	LC467145
	* Cladiella sphaerophora *	MUFS-COAK1	2012.6.5	<3	LC467084	LC467146
	* Klyxum okinawanum *	MUFS-COAK5	2012.6.5	<3	LC467085	LC467147
	*Klyxum* sp.	MUFS-COMO150	2013.7.30	<10	LC467086	NA
		MUFS-COMO164	2013.7.30	<10	LC467087	NA
		MUFS-COOTUD8	2014.12.3	<15	LC467088	NA
	*Sarcophyton* sp.	MUFS-COAK7	2012.6.5	<3	LC467089	NA
		MUFS-COSU16	2012.5.5	<1	LC467090	NA
	*Sinularia* sp.	MUFS-COAK2	2012.6.5	<3	LC467091	NA
		MUFS-COAK3	2012.6.5	<3	LC467092	NA
		MUFS-COAK4	2012.6.5	<3	LC467093	NA
		MUFS-COAK8	2012.6.5	<3	LC467094	NA
		MUFS-COAK9	2012.6.5	<3	LC467095	NA
Nephtheidae	* Dendronephthya rigida *	MUFS-COSS4	2012.5.29	<5	LC467096	NA
	* Dendronephthya gigantea *	MUFS-COSS1	2012.5.29	<5	LC467097	NA
		MUFS-COSS2	2012.5.29	<5	LC467098	NA
		MUFS-COSS3	2012.5.29	<5	LC467099	NA
	* Stereonephthya rubriflora *	MUFS-COSU15	2012.5.5	<1	LC467100	NA
	* Stereonephthya japonica *	MUFS-COAK10	2012.6.6	<10	LC467101	NA

### DNA extraction, amplification, and sequencing

Tissue samples were kept in CHAOS solution for at least a week to dissolve proteins at room temperature. Total DNA was extracted from the CHAOS solution with tissue samples by conventional phenol/chloroform extraction method. We used the primers reported by McFadden et al. (2006) to amplify a fragment 5' end of the mitochondrial NADH-dehydrogenase subunit 2 gene (ND2) (16S647F: 5' -ACA CAG CTC GGT TTC TAT CTA CCA-3'; ND21418R: 5' -ACA TCG GGA GCC CAC ATA-3'). We also used two primers (1S: 5'-GGT ACC CTT TGT ACA CAC CGC CCG TCG CT-3'; 2SS: 5'-GCT TTG GGC GGC AGT CCC AAG CAA CCC GAC TC-3') (Wei et al. 2006) to amplify the internal transcribed spacer (ITS) of the nuclear ribosomal RNA gene. All PCR reactions contained 1 μL of DNA solution, 1.6 μL of 2.5 mM dNTP Mixture, 2 μL of 10X *Ex Taq* buffer, 2 μL of each 10 mM primer, *Ex taq* (TaKaRa) 0.08 μL, and 11.32 μL of sterile distilled water. Amplifications of these markers were performed (GeneQ PCR Thermal Cycler) with the following thermal profile; 35 cycles of 90 sec at 94 °C, 60 sec at 58 °C, 60 sec at 72 °C. Amplified fragments were checked on 1% agarose gel electrophoresis. All the PCR products were subjected to digest excess primers and inactivation of dNTP using Exonuclease I (TaKaRa) and Shrimp Alkaline Phosphatase (TaKaRa). These DNA sequences were determined by ABI3000 using a research contract service (Ltd. FASMAC).

### Sequence alignment and construction of phylogenetic trees

MEGA5 (Tamura et al. 2011) was used to manually align all the DNA sequences and to reconstruct phylogenetic trees. All indels were excluded from the analyses. Molecular phylogenetic trees were reconstructed using Neighbor-joining (NJ) method and maximum-likelihood (ML) method with model parameters (ND2: T92 + G, ITS: K2 + G) with 1000 bootstrap replicates. All the DNA sequences we obtained in this study were registered into DDBJ (accession nos. LC467016–LC467147).

## Results

### Identification of Xeniidae

**Figure 2.**
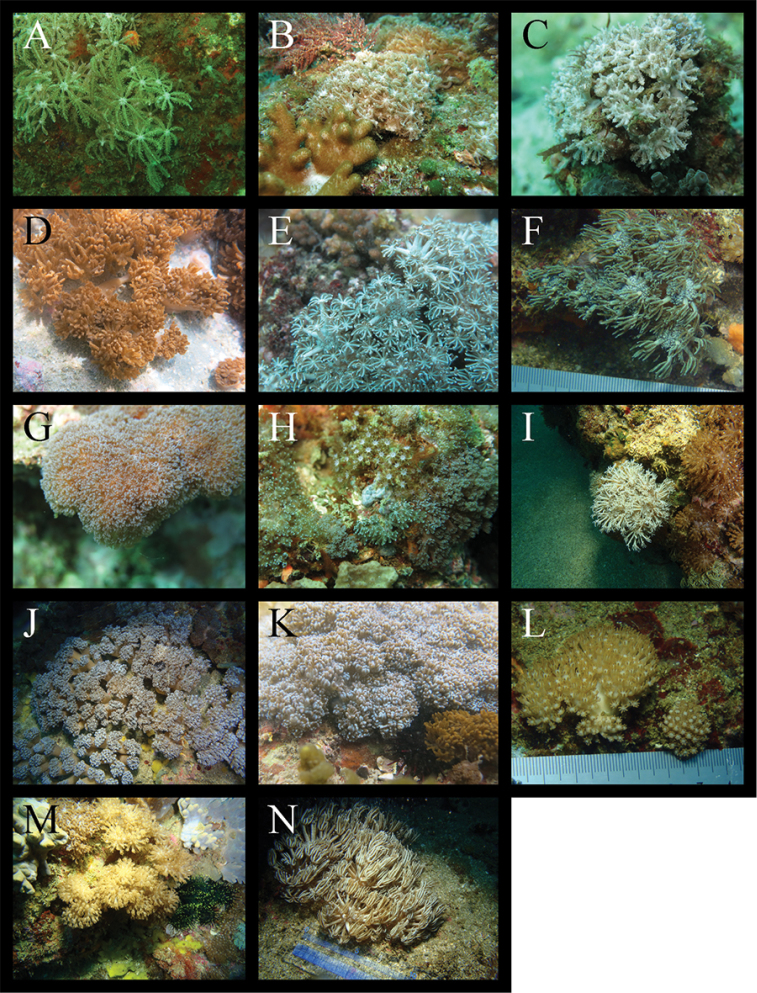
Living form of Xeniidae. **A**Antheliacf.glauca**B***A.rosea***C**A.cf.tosana**D**Heteroxeniacf.elisabethae**E***H.medioensis***F***H.minuta***G***Sympodium* sp. 1 **H***S.* sp. 2 **I***Yamazatum* sp. 1 **J***Xenia* sp. 1 **K***X.* sp. 2 **L***X.novaecaledoniae***M***X.kuekenthali***N***X.plicata*.

**Figure 3.**
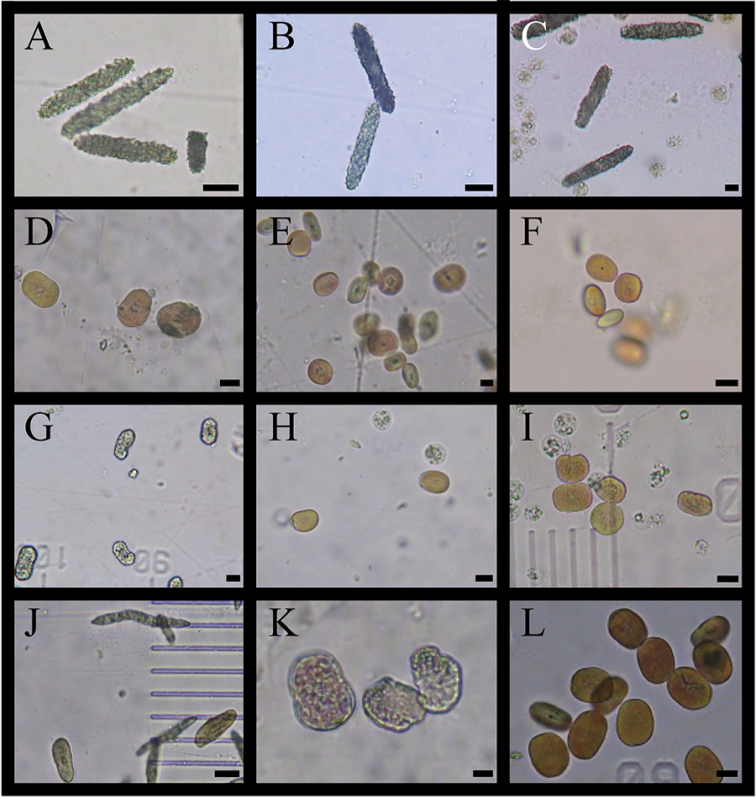
Sclerites of Xeniidae. **A**Antheliacf.glauca**B***A.rosea***C**A.cf.tosana**D**Heteroxeniacf.elisabethae**E***H.medioensis***F***H.minuta***G***Sympodium*. sp. 1 **H***S.* sp 2. **I***Yamazatum* sp. 1 **J***Xenia* sp. 1 **K***X.* sp. 2 **L***X.plicata*. Scale bars: 10 μm.

**Figure 4.**
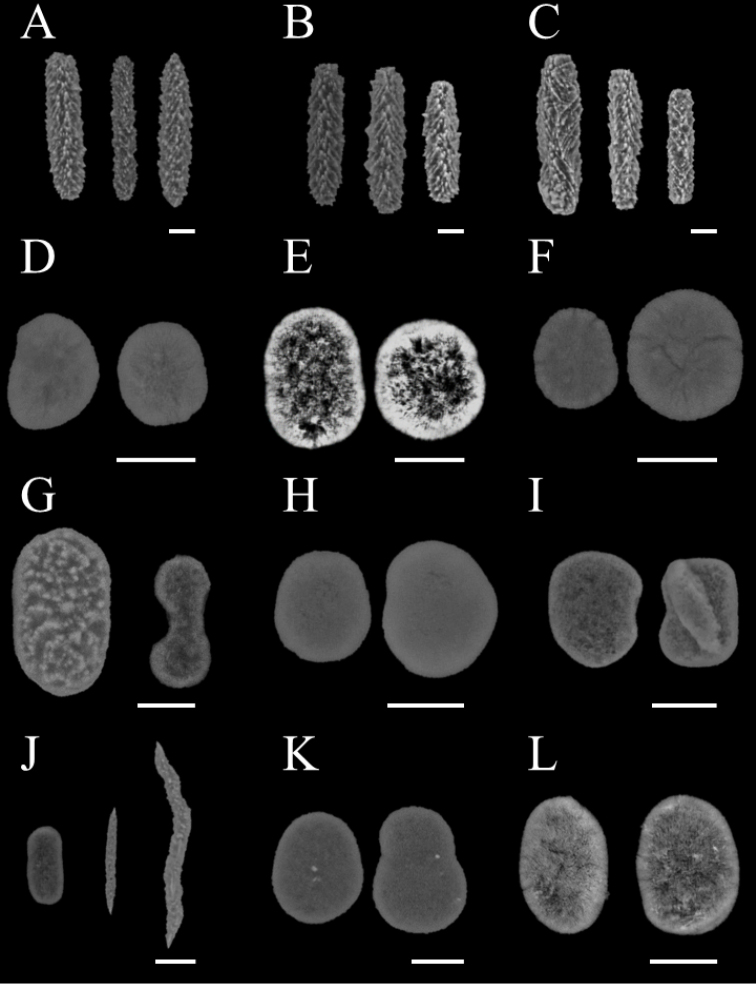
Scanning electron micrographs of sclerites of Xeniidae. **A**Antheliacf.glauca**B***A.rosea***C**A.cf.tosana**D**Heteroxeniacf.elisabethae**E***H.medioensis***F***H.minuta***G***Sympodium*. sp. 1 **H***S.* sp. 2 **I***Yamazatum* sp. 1 **J***Xenia* sp. 1 **K***X.* sp. 2 **L***X.plicata*. Scale bar: 10 μm.

A total of 14 species from five genera in the family Xeniidae were identified: three species from *Anthelia*, three from *Heteroxenia*, two from *Sympodium*, five from *Xenia*, and one species from *Yamazatum* (Table 1). Since inconsistencies were found between the taxonomic morphological characteristics of some specimens and those of species described previously, those specimens were temporarily treated as either unidentified species (e.g., *Xenia* sp. 1), or closely related to specific species (e.g., Heteroxeniacf.elisabethae). Figures 2 and 3 show underwater photographs and optical microscope images of those species’ sclerites. Among these, 12 species (*Xenianovaecaledoniae* Verseveldt, 1974, *X.* sp. 1, *X.* sp. 2, *Yamazatum* sp. 1, *Sympodium* sp. 1, *Sympodium* sp. 2, Heteroxeniacf.elisabethae Kölliker, 1874, *H.medioensis* Roxas, 1933, *H.minuta* Roxas, 1933, Antheliacf.glauca Lamarck, 1816, *A.rosea* Hickson, 1930, A.cf.tosana Utinomi, 1958) were first recorded in Japan. Additionally, we checked these species’ sclerite microstructures (Fig. 4), as these have been used recently in the taxonomy of Xeniidae (Janes and Mary 2012). We observed that three out of five *Xenia* species (*X.plicata* Schenk, 1896, *X.* sp. 1, *X.* sp. 2) exhibited the typical genus microstructure (i.e., dendritic rods) whereas the remaining two species (*X.kuekenthali* Roxas, 1933 and *X.novaecaledoniae*) presented no sclerites. Furthermore, we found that all three *Heteroxenia* species (H.cf.elisabethae, *H.medioensis* and *H.minuta*) exhibited similar microstructures to *Xenia* spp. None of these specimens presented sclerites, comprising aggregations of minute corpuscular-shaped microscleres (Alderslade 2001), which is a specific characteristic of *Ovabunda*, a genus related closely to *Xenia*. Two *Sympodium* species presented a very specific microstructure (see below). *Yamazatum* sp. 1 exhibited the typical sclerite architecture (crests on sclerites’ surface) of this genus (Fig. 4I).

In the present study, *Xenia* sp. 1, *X.* sp. 2, *Yamazatum* sp. 1, *Sympodium* sp. 1 and *S.* sp. 2 were identified as undescribed species for the following reasons: *Xenia* sp. 1 shared common morphological characteristics with the genus *Xenia*, such as the colony shape and the presence of oval sclerities, but presented also with unique needlelike sclerites with many small spines (Fig. 4J), which have never been reported in *Xenia*. *Xenia* sp. 2 was easily distinguishable from other *Xenia* species, as it presented many short branches extending from the top of colony, becoming hump-shaped (Fig. 2K). *Yamazatum* is a monotypic genus containing *Y.iubatum* Benayahu, 2010 and presenting two specific morphological characteristics: doubleheaded sclerites and a conspicuous crest on the sclerites’ surface. *Yamazatum* sp. 1 presented a crest on the sclerites’ surface (Fig. 4I) but lacked doubleheaded sclerites; in this species the sclerites were found only in the polyps, opposite to *Y.iubatum*, containing sclerites both in the surface and interior of the stalk layer and in the polyps. Furthermore, this species presents a branching stalk (Fig. 2I), opposite to *Y.iubatum*, which has a non-branching stalk. *Sympodium* sp. 1 and *S.* sp. 2 shared the common morphological characteristics of the genus *Sympodium*, such as a thin stolon-like sheet and no stalks in colony (Fig. 2G, H). However, both species found in this study presented unique sclerites, which differed from all eight known *Sympodium* species. *Sympodium* sp. 1 presented two types of sclerites; one a doubleheaded sclerite, typical from *Y.iubatum*, located in the polyps (Fig. 4G), and an oval sclerite with protrusions like a mountain range, located on the coenenchyme (Fig. 4G). *Sympodium* sp. 2 presented disk-shaped sclerites throughout the whole colony, with smooth surfaces and no protrusions (Fig. 4H). Under a light microscope the sclerites of *Sympodium* sp. 1 were mostly colorless, whereas those of *Sympodium* sp. 2 were light brown (Fig. 3G, H).

### Molecular phylogenetic analyses

**Figure 5.**
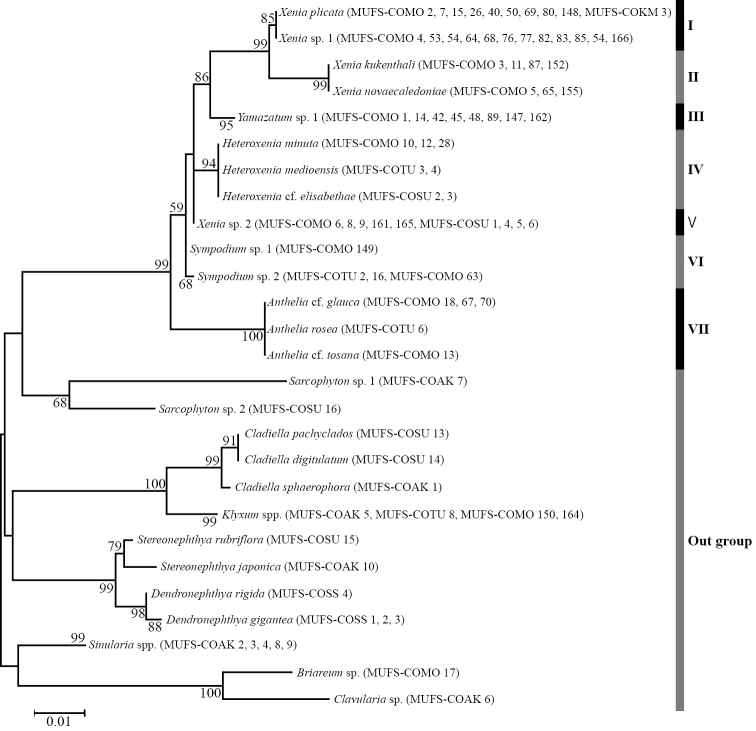
Phylogenetic relationships of species in Xeniidae based on ND2 sequences. Numbers on main branches show percentages of bootstrap values (> 50%) in maximum likelihood analysis.

**Figure 6.**
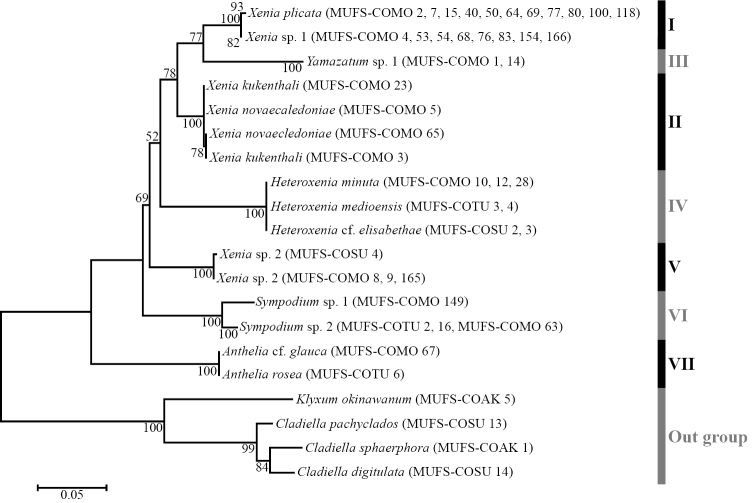
Phylogenetic relationships of species in Xeniidae based on ITS sequences. Numbers on main branches show percentages of bootstrap values (> 50%) in maximum likelihood analysis.

From the collected 14 species (78 samples), we obtained 673–707 bases of ND2 and 910–1039 bases of ITS. Molecular phylogenetic trees using the NJ and ML methods showed very similar topologies. Therefore, in this study, only ML trees for each marker are shown (Figs 5, 6). These trees showed that the family Xeniidae was monophyletic in the Alcyonacea, and that the xeniid species were separated into seven clades. Clade I included *Xeniaplicata* and *X.* sp. 1. Although the ND2 tree showed an absence of genetic differences between these two species (Fig. 5), the ITS tree showed that they were clearly separated from each other (Fig. 6). Clade II included *X.kuekenthali* and *X.novaecaledoniae*, and clade III included only one species, *Yamazatum*. sp. 1. The ND2 tree showed that clade III formed a sister group with clades I and II with *Xenia* spp., whereas the ITS tree showed that clade III formed a sister group with only clade I. Clade IV contained all three *Heteroxenia* species (H.cf.elisabethae, *H.medioensis*, *H.minuta*). Clade V contained a single species *X.* sp. 2. Clades VI and VII contained *Sympodium* spp. and *Anthelia* spp., respectively. Thus, four genera (*Anthelia*, *Heteroxenia*, *Sympodium*, and *Yamazatum*) were monophyletic (clades III, IV, VI, VII) whereas *Xenia* was polyphyletic (clades I, II, V) because clades III and IV with *Heteroxenia* and *Yamazatum* were included within clades of *Xenia*.

### Comparison between phylogenetic relationships and morphological characteristics

In the present study, except *Xenia*, all genera were monophyletic (clades III, IV, VI, VII). Therefore, the synapomorphy reflecting each of the four clades is consistent with the key morphological characteristics for each genus. On the other hand, only *Xenia* was polyphyletic (clades I, II, and V). Therefore, to determine the synapomorphy for each clade, the morphological characteristics of the species in these three clades were compared. In clade I, including *X.plicata* and *X.* sp. 1, the synapomorphy is a colony form 25–40 mm in height and without secondary branches. Clade II, including *X.novaecaledoniae* and *X.kuekenthali*, presented a colony form similar to clade I (typical and no secondary branches), but shorter (10–20 mm in height). It is noteworthy that, although the family Xeniidae is taxonomically defined as presenting oval sclerites, both species in clade II lacked sclerites. Clade V, with just *X.* sp. 2, was characterized by a unique colony form, comprising a stalk measureing about 10 mm high and 20 mm in diameter, and many short branches extending from the top, becoming hump-shaped. This type of colony form has not been reported previously in the genus *Xenia*.

## Discussion

### High species diversity in the family Xeniidae in Miyazaki

The present study identified 14 species from five genera in the family Xeniidae around Oshima Island, Miyazaki, in Japan. Among these species, 12 (Antheliacf.glauca, *A.rosea*, A.cf.tosana, H.cf.elisabethae, *H.minuta*. *H.medioensis*, *Sympodium* sp. 1, *S.* sp. 2, *Xenianovaecaledoniae*, *X.* sp. 1, *X.* sp. 2 and *Yamazatum* sp. 1) were recorded in Japan for the fitst time, including five undescribed species (*Sympodium* sp. 1 and *S.* sp. 2, *Xenia* sp. 1, *Xenia* sp. 2 and *Yamazatum* sp. 1). On the other hand, two genera, *Fungulus* Tixier-Durivault, 1970 and *Cespitularia*, recorded previously in Japan (Utinomi 1977; Imahara 1991; Benayahu 1995, 2010) were not found in Oshima Island.

Miyazaki has the highest Xeniidae species diversity in Japan (Table 2; Suppl. material 6: Table S6). Taking together the results from the present study and those from two previous reports (Imahara 1996; Benayahu 2010), eight genera and 32 species have been confirmed in Japan, the fourth highest Xeniidae diversity in the world (Table 2). Considering that the top three regions are tropical coral reef regions (Philippines, Red Sea, and Indonesia), Xeniidae has a relatively higher species diversity in Japanese waters than in the other regions listed in Table 2, despite its higher latitude. One reason behind this may be the larval supply from the tropics, brought by the strong warm Kuroshio Current that flows from the Philippines (with many coral reefs) up to Kyushu Island including Oshima Island, and the mainland of Japan.

**Table 2.** Distribution of Xeniidae by country. List of number of species and genera of the family Xeniidae, previously reported in the world. See Suppl. material 6: Table S6 for reference numbers in referece.

Location	Number of species	Number of genera	References
Philippines	42	5	15, 16, 26, 27, 29, 32
Indonesia	38	7	2, 12, 13, 16, 18, 21, 26, 28, 30, 32, 36
Red Sea	35	6	3, 10, 11, 12, 13, 16, 21, 24, 25, 26, 28, 30, 39, 40, 41, 44, 45, 46
Japan	32	8	4, 5, 14, 15, 16, 23, 32, 34, 35, 37, This study
Miyazaki Prefecture	14	5	This study
Nansei Islands	13	7	4, 5, 14, 15, 16, 37
Mainland of Japan (Honshu, Shikoku and Kyushu)	8	3	16, 23, 32, 34, 35
Australia	27	8	1, 13, 15, 16, 21, 30, 43
Tanzania	17	6	13, 16, 21, 30, 31
Taiwan	13	6	7, 8, 16, 32, 33
Seychelles	11	5	12, 17, 18
New Caledonia	7	3	15, 16, 18, 21, 42
Mozambique	6	4	13, 18, 21, 31, 32
Papua New Guinea	6	2	13, 21, 30, 31, 47
Fiji	6	2	1, 13, 21, 28
Palau	4	3	16, 18, 32
Malay	4	3	1, 16
Chagos Archipelago	4	3	1, 12, 21, 26, 31
Cargados Carajos	3	3	31
Norwegian Sea	3	2	9, 19, 20
Madagascar	2	2	12, 16
Kenya	2	2	30
Tonga	2	2	13, 21, 28
Republic of South Africa	2	2	21, 22
Sri Lanka	2	1	13
Singapore	1	1	6
Samoa	1	1	28
New Zealand	1	1	11
Hong Kong	1	1	38
Korea	1	1	16, 21, 31
Antarctic Ocean	1	1	13, 21, 22
Guam	1	1	18

### Ecological succession in temperate coral communities

Alcyonacean corals (soft corals) have been known as pioneers in coral reefs (Benayahu and Loya 1987; Fabricius 1995), as well as negative indicators of the early developmental processes of the zooxanthellate scleractinian corals (Maida et al. 1995, 2001). Thus, alcyonacean corals play an important role for ecological succession in coral reefs. Around Oshima Island, zooxanthellate scleractinian corals were dominant until the 1980s, probably representing the late stage of ecological succession in the coral community. Subsequently, these corals were damaged by *Drupella* spp. and *Acanthaster* sp. (Takayama and Shirasaki 1990). Currently, many zooxanthellate alcyonacean corals inhabit the top of dead coral skeletons, which may represent the initial stage of the secondary ecological succession in this coral community. In fact, Endean (1976) reported that Alcyonacea attached onto dead coral skeletons after feeding damage by *Acanthaster* sp. One of the most dominant alcyonacean corals in Oshima Island is Xeniidae, which may be related to its faster growth, rapid colony migration and asexual reproduction (Benayahu and Loya 1985). Although no species diversity data pertaining to hard and soft corals are currently available from the time when hard corals were dominant, the fact that the three-dimensional structures constructed by the zooxanthellate scleractinian corals are gone, suggests that the biota in Oshima Island might have been dramatically different than the present one. Therefore, it would be worthwhile to continuously investigate the change of biota in this area, to understand the process of ecological succession of the benthic and coral community at this higher latitudinal region.

### Phylogeny and taxonomy of the Xeniidae

*Heteroxenia* and *Yamazatum* were monophyletic, although *Xenia* were closely related to both genera (Figs 5, 6). Although *Heteroxenia* presents dimorphic polyps composed of autozooids (normal polyps) and siphonozooids (i.e., no tentacles in polyps, but functional for inhalation and discharge of seawater), siphonozooids only develop when the colony is sexually mature (Gohar 1940; Fabricius and Alderslade 2001). Thus, *Heteroxenia* and *Xenia* can only be superficially distinguished during the breeding season, since during the non-breeding season *Heteroxenia* contains one type of polyp only (autozooids). The present study shows that *Xenia* and *Heteroxenia* can be clearly separated in the molecular trees, although some colonies of *Heteroxenia* were found not to form siphonozooids. These colonies were morphologically identified as *Heteroxenia*, based on the colony size and shape, the autozooids, the pinnules and the sclerites, despite the occurrence of dimorphic polyps. Although the presence or absence of siphonozooids, an important morphological characteristic for Alcyonacea’s generic classification, was confirmed for *Xenia* and *Heteroxenia*, molecular phylogenetic analyses of all the 11 species of *Heteroxenia* are necessary to properly define the taxonomic position of this genus.

In the present study, the phylogenetic position of *Yamazatum* sp. 1 was ambiguous as this species formed a sister group with clade I in the ND2 tree (Fig. 5), and with both clades I and II in the ITS tree (Fig. 6). Currently, several xeniid genera, including *Yamazatum* are taxonomically classified based only on sclerite surface microstructure (*Bayerxenia* Alderslade, 2001; *Ingotia* Alderslade, 2001; *Ixion* Alderslade, 2001; *Orangaslia* Alderslade, 2001; *Ovabunda*; *Fasciclia* Janes, 2008; *Conglomeratusclera* Benayahu et al., 2018; *Caementabunda* Benayahu et al., 2018; and *Yamazatum*). Although most of these genera have never been analyzed molecularly, a recent molecular phylogenetic analysis revealed that *Ovabunda* belonged to the same clade as *Xenia* (Haverkort-Yeh et al. 2013; McFadden et al. 2014), which, in the present study, is also in the clade of *Yamazatum*. Therefore, detailed comparisons between molecular data and the sclerite microstructure will be needed for future xeniid taxonomic classification.

**Figure 7.**
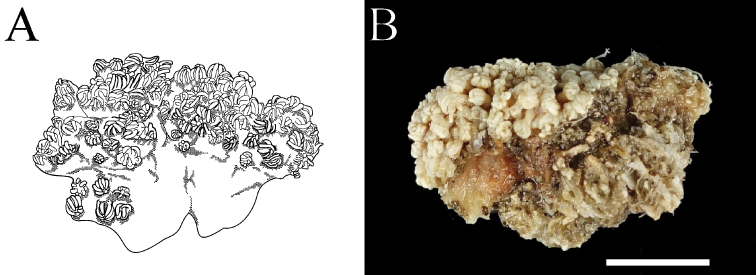
*Xenia* sp. 2. **A** schema of *Xenia* sp. 2 **B** photo of a specimen of *Xenia* sp. 2 (MUFS-COMO9). Scale bar: 10 mm.

*Xenia* was polyphyletic, particularly due to *X.* sp. 2 (Figs 5, 6). Clade V with *X.* sp. 2 was closer to clade IV with *Heteroxenia* than other *Xenia* clades (clades I and II). *Xenia* sp. 2 exhibited slight but substantial differences from its congeners in terms of colony morphology, as their colony shapes lacked branching, exhibiting dome-shaped protrusions (Fig. 7). Considering that *Heteroxenia* presents specific characteristics that distinguish it from *Xenia*, such as dimorphic polyps, the species *X.* sp. 2 may be assigned to a new genus, although this requires further investigations into the morphological characteristics of other genera not observed in present study.

Two undescribed species, *S.* sp. 1 and *S.* sp. 2, were found in *Sympodinium*, and presented different sclerites and microstructure types (Fig. 4) from their congeners. Currently, this genus has only eight species, *S.abyssorum* Danielssen, 1887, *S.caeruleum* (Ehrenberg, 1834), *S.fuliginosum* Ehrenberg, 1834, *S.hyalinum* Grieg, 1887, *S.norvegicum* Koren & Danielssen, 1883, *S.punctatum* May, 1898, *S.splendens* Thomson & Henderson, 1906 and *S.tamatavense* (Cohn, 1908). Their type localities are the Red Sea for *S.caeruleum and S.fuliginosum*, Norwegian Sea for *S.abyssorum*, *S.hyalinum* and *S.norvegicum*, Indian Ocean *S.punctatum* and *S.splendens*, and Madagascar for *S.tamatavense*. Except for *S.caeruleum*, all species have never been recorded in the Pacific region, probably due to the lack of research into this genus. Therefore, more species are likely to be found in the Pacific region in the future.

Studies on the species composition and biodiversity of alcyonacean corals have drawn considerably less attention than those on scleractinian corals, since alcyonacean corals do not form the same three-dimensional structures with their hard skeletons as scleractinian corals, and, therefore, provide less habitat for other animals. However, coral communities have been reported to shift from scleractinian corals to alcyonacean corals in the future, if ocean acidification persists (Inoue et al. 2013). Thus, further ecological and taxonomic studies of alcyonacean corals are needed. Although the current taxonomic classification of alcyonacean corals is still underdeveloped, this may be improved by further molecular analyses and accurate species identification will improve this situation.

## Supplementary materials

Supplementary material 1 Table S1. Morphological features and key characters for species identification of *Xenia* Data type: morphological data This dataset is made available under the Open Database License (http://opendatacommons.org/licenses/odbl/1.0/). The Open Database License (ODbL) is a license agreement intended to allow users to freely share, modify, and use this Dataset while maintaining this same freedom for others, provided that the original source and author(s) are credited.Tatsuki Koido, Yukimitsu Imahara, Hironobu Fukami

Supplementary material 2 Table S2. Morphological features and key characters for species identification of *Yamazatum* Data type: morphological data This dataset is made available under the Open Database License (http://opendatacommons.org/licenses/odbl/1.0/). The Open Database License (ODbL) is a license agreement intended to allow users to freely share, modify, and use this Dataset while maintaining this same freedom for others, provided that the original source and author(s) are credited.Tatsuki Koido, Yukimitsu Imahara, Hironobu Fukami

Supplementary material 3 Table S3. Morphological features and key characters for species identification of *Heteroxenia* Data type: morphological data This dataset is made available under the Open Database License (http://opendatacommons.org/licenses/odbl/1.0/). The Open Database License (ODbL) is a license agreement intended to allow users to freely share, modify, and use this Dataset while maintaining this same freedom for others, provided that the original source and author(s) are credited.Tatsuki Koido, Yukimitsu Imahara, Hironobu Fukami

Supplementary material 4 Table S4. Morphological features and key characters for species identification of *Sympodium* Data type: morphological data This dataset is made available under the Open Database License (http://opendatacommons.org/licenses/odbl/1.0/). The Open Database License (ODbL) is a license agreement intended to allow users to freely share, modify, and use this Dataset while maintaining this same freedom for others, provided that the original source and author(s) are credited.Tatsuki Koido, Yukimitsu Imahara, Hironobu Fukami

Supplementary material 5 Table S5. Morphological features and key characters for species identification of *Anthelia* Data type: morphological data This dataset is made available under the Open Database License (http://opendatacommons.org/licenses/odbl/1.0/). The Open Database License (ODbL) is a license agreement intended to allow users to freely share, modify, and use this Dataset while maintaining this same freedom for others, provided that the original source and author(s) are credited.Tatsuki Koido, Yukimitsu Imahara, Hironobu Fukami

Supplementary material 6 Table S6. List of references used in Table 2 Data type: reference data This dataset is made available under the Open Database License (http://opendatacommons.org/licenses/odbl/1.0/). The Open Database License (ODbL) is a license agreement intended to allow users to freely share, modify, and use this Dataset while maintaining this same freedom for others, provided that the original source and author(s) are credited.Tatsuki Koido, Yukimitsu Imahara, Hironobu Fukami
